# Measuring the psychological burden of women with pelvic floor complaints: The psychometric characteristics of a new instrument

**DOI:** 10.12688/openreseurope.15833.1

**Published:** 2023-05-17

**Authors:** Alma Brand, Wim Waterink, Scott Rosas, Jacques van Lankveld

**Affiliations:** 1 Psychology, Open Universiteit, Heerlen, Limburg, 6419 AT, The Netherlands; 2 Concept Systems Inc., Ithaca, New York, NY 14850, USA

**Keywords:** Psychological burden, Pelvic floor complaints, Inventory, Scale development, Scale validation, Psychometric analyses, Valid Instrument, Reliable measure

## Abstract

**Background**: To be able to optimize pelvic healthcare, it would be helpful to specifically assess women’s psychological burden with pelvic floor complaints. In the absence of such an instrument, a new instrument was developed to measure this burden in women who seek help. In previous research, a comprehensive overview was yielded of women’s restrictions and distress with pelvic floor complaints, and a conceptual model was developed of seven types of distress that were reflected by 33 statements. The present study was performed to investigate the psychometric properties of the new instrument, termed the Pelvic Floor Complaint-related Psychological Burden Inventory (PFC-PBI).

**Methods**: In an online survey data was collected from women with and without pelvic floor complaints on the 33 statements. The internal consistency of the types of distress was tested, Principal Component and Confirmatory Factor Analyses were performed, and the convergent and divergent validity of the types of distress was examined against existing questionnaires.

**Results**: Based on the factor analyses, a 10-item instrument was tested. Outcomes show excellent internal consistency of this instrument, comprising a single component. The PFC-PBI demonstrated satisfactory convergent and divergent validity.

**Conclusions**: This new measure appears to be a promising tool to inventory the psychological burden of women suffering from pelvic floor complaints. Research into its further development, implementation, and clinical use appears warranted.

## Plain language summary

To be able to optimize pelvic healthcare for women, it would help to be able to specifically assess women’s psychological burden with pelvic floor complaints. There is no instrument to measure this burden in women who seek help. Previous research has clarified this burden in a model that reflects seven types of distress and provided 33 statements that represent this burden. This study investigated which statements were most representative of women’s psychological burden and that together formed a reliable instrument to assess it. The new instrument is termed the Pelvic Floor Complaint-related Psychological Burden Inventory (PFC-PBI). Women with and without pelvic floor complaints scored these 33 statements in an online survey. Statistical analyses were performed to assess the characteristics and combination of statements that most accurately represent women’s psychological burden with pelvic floor complaints. Based on these analyses, a 10-item instrument was tested. The outcomes were excellent, and the PFC-PBI was found valid and reliable. This new instrument appears to be a promising tool to inventory the psychological burden of women suffering from pelvic floor complaints. Research into its further development, implementation, and clinical use appears justified.

## Introduction

Urinary and fecal incontinence, micturition and defecation problems, pelvic organ prolapses, pelvic pain, and painful intercourse are pelvic floor complaints (PFC) that are prevalent in women ^1– 8^. The prevalence of PFCs has been found to be higher during pregnancy and after childbirth ^9, 10^. The fact that some women with PFC seek help in pelvic physical therapy (PPT) practice while others do not ^3, 9^, has raised questions about predictive factors for seeking help.

Previous research in this field identified restrictions and distress that women with PFC experience in their daily, social, and sexual functioning and their intimate relationships ^1, 3– 6, 11– 19^. These findings suggested that their psychological burden might be an important determinant to seek help. Because the available evidence from previous studies is inconclusive because it mostly focused on specific complaints and specific domains of life, two mixed-method studies were performed among Dutch young adult pregnant, parous, and nulliparous women with PFC ^20, 21^. In the first study, women were interviewed about the restrictions and distress they experienced related to their PFC. The study provided a comprehensive overview of women’s psychological burden with PFC based on text-mining analyses ^20^. In the second study, a conceptual model of women’s psychological burden with PFC was developed using Group Concept Mapping methodology ^21^. Although these studies have added to the understanding of women’s psychological burden with PFC, a valid instrument to measure this burden is not yet available. Drawing from the aforementioned theoretical and conceptual studies, the current study investigated the psychometric properties of the conceptual model.

From the conceptual study, 33 items were derived that represent seven types of distress emerging in the model. These types of distress were *Loss of Control*, *Feeling Insecure*, *Feeling Wronged*, *Feeling Helpless*, *Sexual Distress*, *Feeling Angry*, and *Feeling Disappointed* ^21^. The 33 items were viewed in the present study as candidate items for instrument development and further psychometric testing. This study investigated the psychometric quality of a new instrument for assessing women’s psychological burden with PFC.

## Methods

### Study design

Dutch pregnant, parous, and nulliparous women with and without PFC, aged between 18 and 45 years, participated in the online survey in the present cross-sectional study. Pregnant women were expecting their first baby, parous women had a child younger than two years old, and nulliparous women had no children and were not pregnant. Women were recruited by personal invitation and on social media using targeted invitations and advertisements, with help of pelvic physical therapists, midwives, and general practitioners, Hersenonderzoek.nl ( www.hersenonderzoek.nl) using targeted recruitment through a temporary project on their website which was paid for by the main researcher, and through the social networks of the participating women (snowball method). In general, a subgroup sample size meeting the 10:1 subject-to-variable (STV) ratio criterion is recommended to maximize the population estimates’ accuracy ^22^. Women who were pelvic physical therapists themselves were excluded from participation, as were women without PFC who sought help. Based on experience from clinical practice, some women without PFC seek help for the purpose of prevention.

### Data preparation and collection

In the survey, demographic questions, questions to assess women's eligibility for participation, and questions about women’s present and past treatment in PPT were asked. The presence of seven common PFC was inventoried based on their occurrence during the past month, using the most representative items for each individual complaint from the Pelvic Floor Distress Index (PFDI) ^23^. *Urinary Incontinence* (UI; 2 items), *Fecal Incontinence* (FI; 2 items), *Micturition Problems* (MP; 3 items), *Defecation Problems* (DP; 3 items), and *Pelvic Organ Prolapse* (POP; 1 item) were identified. The absence of a complaint was indicated by the options ‘not present’ and ‘no bother’ (score 0), and all other scores indicated presence (score 1). *Pelvic Pain* (PP), including low back and coccyx pain, was identified using 1 item from the Four-Dimensional Symptom Questionnaire (4DSQ) ^24^. Answering options ‘not present’ and ‘sometimes’ indicated PP absence (score 0), and all other scores indicated presence (score 1). To identify *Painful Intercourse* (PI), 1 item of the Female Sexual Functioning Index (FSFI) ^25^ was used. Responses were reverse-scored to align them with the other PFC scores. Answering options ‘never’ indicated PI absence (score 0), and all other scores indicated presence (score 1). The number of PFC was indicated by the sum of all seven presence scores, resulting in a score range from ‘0’ to ‘7’.

Women’s psychological burden was questioned using the previously selected statements from the conceptual model ^21^, which constituted a preliminary item pool. Participants were asked to answer the applicability of the provided 33 items as follows: “Please click on the dot that represents the applicability of this statement to you as best as possible whilst keeping your physical complaints in mind”. Participants scored these statements on a 7-point Likert scale (0 “not applicable” to 6 “fully applicable”). Purposely, no labels were given to scoring options 1 to 5. The statements were offered in random order. The new instrument will be further referred to as the Pelvic Floor Complaint-related Psychological Burden Inventory (PFC-PBI).

To investigate the convergent and divergent validity of the PFC-PBI, validated questionnaires measuring neighboring and non-neighboring distress constructs were included in the survey. General health issues were inventoried using the Four-Dimensional Symptom Questionnaire (4DSQ) ^24^ to examine the divergent validity of the PFC-PBI with the four sub-scales of this measure: general distress, depression, anxiety, and somatization. To examine the PFC-PBI’s convergent validity with pelvic floor complaint-related distress and with distress related to sexual problems, the Prolapse/Incontinence Sexual Questionnaire (PISQ) ^26, 27^ and the Female Sexual Distress Scale (FSDS) ^25^ were used.

### Data collection procedures

The study protocol was approved by the Ethical Review Board of the Open University of the Netherlands on May 29th, 2019/No. U2019/03973/HVM. The participants could access the general information about the study on the online data acquisition portal O4U, read the online informed consent form, and accept it when agreeing to participate. They then received a registration link by email, to anonymously access the survey using a self-generated password and fill out the questionnaires in approximately 20 minutes. The to-be-validated instrument was novel, the other questionnaires were valid and reliable instruments. The survey was tested before the online launch to check all settings, the clarity of instructions, and the functionality of the links to the tasks before commencing the study. A copy of the questionnaire can be found under *Extended* data ^28^. Data collection occurred over a period of 16 months between November 2021 and March 2023.

### Ethical considerations

The study protocol was approved by the Ethical Review Board of the Open University of the Netherlands on May 29th, 2019/No. U2019/03973/HVM. Participants gave informed written consent and were assured of anonymous use and publication of the data they provided.

### Data analysis

Data were analyzed using Jamovi, version 2.3.18 ^29^. Data of participants with missing PFC-PBI scores and participants without PFC were excluded from further analyses. Subsequently, item response distributions were examined based on histograms checking the data for any potential distribution imbalance. Items were removed when more than 80% of participants gave the same answer ^30^. To enable cross-validation, the data set was randomly split into two stratified subsamples of participants with PFC by first splitting the file into pregnant, parous, and nulliparous women to be able to include equal numbers in each subsample. Subsequently, all odd numbers were selected for the PCA and all even numbers for the CFA analysis.

***Internal consistency*.** To assess the reliability of the items included in the new scale, item-total correlations analysis was performed, using the data on the original 33 items ^21^. In the first step items with a negative correlation were deleted. Next, items were excluded in an iterative process, applying the .70 correlation criterion after each round until no correlations < .70 were present ^31^. Both Cronbach’s alpha and McDonald’s omega were calculated ^32, 33^.

***Principal component analysis*.** Principal Component Analysis (PCA) was performed on the first subset of data. To identify the number of relevant components, the scree plot was evaluated and the Eigenvalue > 1 criterion was used. In case multiple factors would be found, Oblique Promax rotation (Kappa = 4) was applied because components were expected to be intercorrelated ^34^. Items were removed when the minimum loading of an item was lower than .32, or when it cross-loaded with other items with loadings higher than .32 ^35^. A significant outcome of the KMO and Bartlett’s test was required. Next, the internal consistency of the components was re-examined for the remaining items using Cronbach’s alpha and McDonald’s omega ^30, 31^.

***Confirmatory factor analysis*.** The CFA was performed on the remaining subset of data to examine the adequacy of the PCA outcomes in a new sample ^37, 37^. Z-statistics and *p*-values were assessed to evaluate the contribution of each item to the model, with higher Z-values indicating a larger contribution of the items to the model ^29^. Subsequently, the goodness of fit of the model with the data was evaluated, using the Comparative Fit Index (CFI), the Tucker-Lewis Index (TLI), and the Root Mean Square Error of Approximation (RMSEA). The model was regarded adequate when CFI³ .90, TLI³ .90, and RMSEA between .05 and .08 with a 90% confidence interval, and good when the CFI > 0.95, TLI > 0.95, and RMSEA < .05 ^29^.

***Convergent and divergent validity*.** The PISQ scores were reverse-coded to align them with the other scores. Correlations between the PBI scores, and scores on the selected validation measures were considered to reflect moderate to strong convergent validity when they were higher than .40, and divergent validity when lower than .40 ^38^. More precisely, the correlations were derived from bivariate correlation analysis in combination with PCA employing oblique Promax rotation (Kappa = 4) based on parallel analyses, to establish convergent and divergent validity using the Multi-Trait Multi-Method methodology (MTMM) ^39, 40^. Table 1 provides an overview of the measures used in the construct validation process.

**Table 1.** Measures in the construct validation of the pelvic floor complaint-related psychological burden inventory.

Instrument	Construct	Answer Options	Subscales	Scoring	Psychometric Information
**4DSQ** Four-Dimensional Symptom Questionnaire (N = 50)	General physical complaints and distress	5-point Likert Scale (Presence)	Distress (16) Somatization (6) Anxiety (12) Depression (16)	**Sum score** per subscale Total of all subscales Max. score: 100 Higher scores represent more symptoms	***Cronbach’s alfa*** Distress: .94 Somatization: .84 Anxiety: .88 Depression: .94
**PISQ-12** Pelvic organ prolapse/ Incontinence Sexual Questionnaire (N = 12)	Sexual distress related to PFC	5-point Likert Scale (Presence)	Behavioral-Emotional distress (4) Physical distress (5) Partner Related distress (3)	**Sum score**	***Cronbach’s Alfa*** .61 to .87
**FSDS** Female Sexual Distress Scale (N = 12)	Distress related to sexual problems	5-point Likert Scale (Presence)	Sexual distress (12)	**Sum score** Max. score: 48 Higher scores represent more sexual dissatisfaction	***Cronbach’s alfa*** .93

## Results

Five hundred and fifty-two Dutch women with PFC provided data. The 59 pregnant, 210 parous, and 283 nulliparous participants were aged between 18 and 45 years old. The mean age in the total sample was 31.2, in the PCA sample 31.4, and in the CFA sample 30.9. The mean gestation age of pregnancy was 25.9 weeks in the total sample, 26.2 weeks in the PCA sample, and 25.6 weeks in the CFA sample. The mean number of children of parous women in the total sample was 1.55, in the PCA sample 1.52, and in the CFA sample 1.57. The mean number of PFC in the total sample was 3.02, in the PCA sample 2.99, and in the CFA sample 3.04. The full dataset can be found under *Underlying data* ^45^.

### Internal consistency

No items qualified for removal based on the evaluation of the response distributions. The iterative process in the reliability analyses on data of the total sample is shown in Table 2. Twenty-three items were removed based on negative and < .70 item-total correlations, resulting in a scale containing 10 items in total. Cronbach’s Alpha and McDonald’s coefficient omega were calculated. The internal consistency was found to be excellent.

**Table 2.** Reliability in the Total, PCA, and CFA sample.

		Total Sample					PCA Sample	CFA Sample
Item		Round 1	Round 2	Round 3	Round 4	Round 5	Odds	Evens
Loss of Control								
1	I am constantly aware of my physical limitations	-.122						
3	I lack control over my body	.584						
**7**	I feel hesitant when I cannot do what I want because of physical reasons	.740	.739	.734	.740	.742	.740	.742
**15**	It is tiring to constantly balance my activities	.796	.798	.800	.804	.799	.788	.810
**17**	I feel tired because of pain	.713	.727	.732	.736	.735	.707	.759
19	I am fearful to push boundaries and as a result, being unable to do anything	.645						
25	I have to think ahead to plan my activities well	.635						
**32**	I struggle with whether I should take the risk of provoking my physical complaints or not	.749	.763	.769	.771	.771	.769	.775
Feeling Insecure								
5	I lack trust in my body	.684						
12	I doubt if what I do is right	.615						
22	I feel it is a sign of weakness to show that I am not well	.642						
29	I feel stressed about the future	.694						
Feeling Wronged								
11	I feel insecure when women in a similar situation can do things that I cannot do	.723	.689					
**16**	I feel sad to have to say I am not well	.761	.741	.727	.720	.711	.687	.740
18	I crawl into my shell when I talk about my physical complaints	.701	.686					
23	I feel lonely, home alone in my own small world	.513						
Feeling Helpless								
4	It is challenging to talk about restrictions that I experience	.689						
8	I feel irritation over other people’s remarks regarding physical complaints	.697						
14	I feel sad that others do not understand	.742	.729	.714	.692			
**21**	I find it difficult that people underestimate my physical complaints	.767	.770	.768	.760	.749	.751	.748
26	I have to defend myself when I have to explain how I suffer	.689						
27	I refuse to participate in social activities with friends due to physical complaints	.625						
30	I feel sad about other people’s judgments	.717	.705	.699				
Sexual Distress								
2	I feel disappointed about the restrictions in my sex life due to physical complaints	.627						
9	I feel helpless about the influence of my physical complaints on my sex life	.688						
13	I feel sad about the things that I am incapable of doing during sex due to physical complaints	.648						
Feeling Angry								
**20**	I feel angry about the severity of my physical restrictions	.785	.793	.796	.801	.806	.806	.809
**24**	I feel helpless because I cannot do what I want	.818	.807	.811	.811	.813	.788	.836
28	I feel shocked when my physical complaints suddenly occur	.634						
**31**	I am furious about the relapses of my physical complaints when I work so hard to recover	.780	.794	.795	.796	.796	.774	.822
**33**	I feel angry when I lack control over my body	.761	.765	.766	.766	.772	.738	.803
Feeling Disappointed								
6	I am going in circles of fear and pain	.651						
10	I feel uneasy when my mobility is limited	.689						
	**Cronbach’s Alfa**	.966	.953	.949	.947	.944	.939	.948
	**McDonald's Omega**	.968	.953	.950	.947	.945	.940	.950

Note: *N* = 552. For PCA *N* = 276. For CFA *N* = 276. The 10 selected pelvic floor complaint-related psychological burden items were included in the reliability analysis of the PCA and CFA samples.

### Principal component analysis

 The PCA of the remaining 10 items produced a scree plot revealing one component with an eigenvalue of 6.492. The KMO measure of sampling adequacy was .938, and Bartlett’s test of sphericity was significant, c ^2^ = 1950, *df* = 45, *p* < .001. The internal consistency of the emergent component and the remaining items after PCA is shown in the middle column in Table 2, showing excellent outcomes. The PCA pattern matrix and descriptive and normality statistics are shown in Table 3.

**Table 3.** Pattern Matrix, Descriptive and Normality Statistics of the Pelvic Floor Complaint-related Psychological Burden Inventory.

Item	Pelvic Floor Complaint-related Psychological Burden Inventory	Principal Component Analysis Sample (N = 276)					Confirmatory Factor Analysis Sample (N = 276)				
		Component Loadings	Mean	SD	Skewness	Kurtosis	Z-Value ( *p* < .001)	Mean	SD	Skewness	Kurtosis
**7**	I feel hesitant when I cannot do what I want because of physical reasons	**0.794**	1.77	1.95	0.69	-0.89	**14.9**	1.92	1.99	0.55	-1.11
**15**	It is tiring to constantly balance activities	**0.833**	1.97	2.04	0.59	-1.04	**16.5**	2.11	2.05	0.52	-1.08
**16**	I feel sad to have to say I am not well	**0.736**	2.09	2.04	0.52	-1.15	**14.7**	2.38	2.12	0.28	-1.37
**17**	I feel tired because of pain	**0.764**	1.34	1.87	1.22	0.15	**15.0**	1.62	1.99	0.91	-0.61
**20**	I feel angry about the severity of my physical restrictions	**0.851**	1.27	1.84	1.20	0.05	**17.0**	1.31	1.75	1.15	0.12
**21**	I find it difficult that people underestimate my physical complaints	**0.802**	1.48	1.91	1.04	-0.26	**14.8**	1.83	2.15	0.71	-1.03
**24**	I feel helpless because I cannot do what I want	**0.834**	1.53	1.95	1.00	-0.42	**17.7**	1.68	1.97	0.85	-0.66
**31**	I am furious about the relapses of my physical complaints when I work so hard to recover	**0.824**	1.41	1.91	1.15	-0.04	**17.6**	1.36	1.86	1.17	0.10
**32**	I struggle with whether I should take the risk of provoking my physical complaints or not	**0.818**	1.31	1.83	1.19	0.16	**16.0**	1.36	1.80	1.11	-0.02
**33**	I feel angry when I lack control over my body	**0.794**	1.50	1.93	1.09	-0.17	**17.1**	1.62	1.95	0.93	-0.47
	***Eigenvalue***	***6.492***									
	***% Of Explained Variance***	***64.92***									

### Confirmatory factor analysis

To test the invariance of the factor structure, CFA was performed on the data of the remaining 276 participants. The single emergent PCA component met the required VTF (3:1) ratio ^41^. The STV ratio of (276/10) 27:1 proved to be satisfactory. The Z-values of the 10 included statements were all positive and significant ^42^. The overall model was significant, c2 = 137, *df* = 35, *p* < .001. The CFI = .954 and TLI = .941 indicated an acceptable to good fit. The RMSEA indices were .103, 90% CI: .085 - .121 indicating a less acceptable fit. The internal consistency of the ten items in the CFA sample was evaluated and proved to be excellent, similar to the PCA sample, as is shown in the right column of Table 2. The CFA pattern matrix and descriptive and normality statistics are shown in Table 3.

### Convergent and divergent validity

Because women were allowed to fill out the survey in stages and not all women finished the survey, there were missing scores on the FSDS in comparison to the other measures. In addition, the PISQ was only filled out by women with a sexual partner. Correlations were calculated between the sum scores of the ten items of the PFC-PBI with the selected questionnaires. Significant correlations with r > .40 were found between the PFC-PBI and the PISQ, FSDS, and 4DSQ subscales distress and somatization. The outcomes also show convergent validity between the PISQ and the FSDS scores. Furthermore, divergent validity of the PFC-PBI with the 4DSQ subscales of anxiety and depression was found (see Table 4).

**Table 4.** Correlations between the Pelvic Floor Complaint-related Psychological Burden Inventory and measures of neighboring and non-neighboring constructs.

	PFC-PBI (N = 541)	PISQ (N = 432)	FSDS (N = 498)	4DSQ Distress (N = 552)	4DSQ Somatization (N = 552)	4DSQ Anxiety (N = 552)
**PISQ**	.440 ***					
**FSDS**	.497 ***	.650 ***				
**4DSQ** **Distress**	.462 ***	.324 ***	.343 ***			
**4DSQ** **Somatization**	.460 ***	.242 ***	.262 ***	.574 ***		
**4DSQ** **Anxiety**	.377 ***	.142 **	.205 **	.700 ***	.507 ***	
**4DSQ** **Depression** **(N = 552)**	.322 ***	.198 ***	.224 ***	.691 ***	.336 ***	.592 ***

Note: * *p* < .05, ** *p* < .01, *** *p <* .001.

To further explore the discriminant validity of the PFC-PBI the MTMM approach was followed. Therefore, another PCA was performed using Promax rotation with the PBI and the selected validation measures. The outcomes confirm the convergent validity of the PBI with the PISQ and the FSDS, and the divergent validity with the four subscales of the 4DSQ. The KMO measure of sampling adequacy was .782, and Bartlett’s test of sphericity was significant, c2 = 1093, *df* = 21, *p* < .001 (see Table 5).

**Table 5.** Principal Component Analysis of the Pelvic Floor Complaint-related Psychological Burden Inventory subscales and the selected validation measures.

Questionnaires	Component 1	Component 2	Uniqueness
**PFC-PBI**		.619	.405
**PISQ**		.923	.245
**FSDS**		.910	.223
**4DSQ-Distress**	.871		.181
**4DSQ-Somatization**	.572		.543
**4DSQ-Fear**	.923		.263
**4DSQ-Depression**	.819		.392
***Eigenvalue***	***3.41***	***1.34***	
***% Of Explained Variance***	***48.72***	***19.13***	
***Cumulative %***	***48.7***	***67.8***	

Note: *N* = 552.

## Discussion

This study aimed to evaluate the psychometric properties of a new instrument, the PFC-PBI, to be used to assess women’s psychological burden with PFC. The internal consistency of the selected items was excellent. PCA and CFA were performed using data from stratified (split-half) independent samples to examine the factor structure and to cross-validate this emergent structure in a new sample. The instrument’s homogeneity and convergent and divergent validity were examined including the data from the total sample. In addition, the convergent validity with the PISQ and FSDS, and divergent validity with the 4DSQ subscales were found in line with what was expected. This rendered the PFC-PBI a valid and reliable instrument to uniquely measure women’s psychological burden with PFC.

When comparing the ten remaining PFC-PBI items with the 33 statements and their location in the conceptual model that was based on earlier qualitative research ^20, 21^, four types of distress from the original model were included in the new instrument. The items from the other three types of distress were excluded from the emergent item list of the PFC-PBI. This requires further discussion and clarification.

The first point of discussion regards the *Feeling Disappointed* items. *Feeling Disappointed* was a frequently expressed type of distress by women with PFC regarding restrictions in their daily, social, and sexual functioning, and their intimate relationships in an earlier interview study ^20^. Furthermore, it was included in the conceptual model by women with PFC and health care providers ^21^. However, when exploring the literature on this topic, disappointment appears to be an understudied type of distress in women with PFC ^20^. Results in this study raise questions as to why the *Feeling Disappointed* items were excluded from the PFC-PBI. A plausible reason for this might be found in the meaning of disappointment which is defined as feeling sad or displeased because someone or something has failed to fulfill one’s hopes or expectations. This implies that *Feeling Disappointed* can encompass different modalities such as sadness, anger, and feeling let down which may render it less representative of one particular type of distress. Another reason for its exclusion could be the fact that the *Feeling Disappointed* cluster only contained two statements, rendering it a weaker construct from the start, and that on closer examination of its content, a mismatch with the definition of disappointment was implied. Going in circles of fear and pain, and feeling uneasy about limited mobility does not exactly comply with the disappointment definition. The (dis)appearance of the *Feeling Disappointed* items raises questions about its relevance in the context of PFC which supports the need to further study disappointment in this context.

The second point of discussion regards the items representing *Feeling Insecure.* These items emerged as a pervasive type of distress in the central position of the conceptual model ^21^. Insecurity is mentioned in the literature as a prevalent type of distress with PFC ^9^, and sexual dysfunction ^43^, complaints that often co-occur ^16, 17, 19, 44, 45^. It may be due to their pervasive nature that these items are excluded from the PFC-PBI, rendering them not specific enough to qualify as a true representation of women’s psychological burden with PFC.

Thirdly, the topic of *Sexual Distress* needs to be discussed. Despite the fact that the three included statements from the model cover a clear and coherent take on women’s sexual distress with PFC, they were excluded from the PFC-PBI based on the psychometric analyses in this study. An explanation for this finding could be that they do not fully fit the psychological burden related to all types of PFC that have their specific nature and consequences. Furthermore, several valid and reliable instruments to measure sexual distress already exist, which reduces the need to include them in the PFC-PBI. Despite the exclusion of these items from the PFC-PBI, the new scale shows acceptable convergent validity with the PISQ and FSDS which both cover the topic of sexual distress with pelvic floor complaints and with sexual dysfunction.

However, the exclusion of statements from these three types of distress from the conceptual model could be further examined in a more detailed analysis of the emergent scale in relation to the original theoretical model. Outcomes may clarify why these items were excluded from the PFC-PBI. In addition, the assessment of suitability for its use among pregnant, parous, and nulliparous women specifically could make the PFC-PBI more specific and selective in different circumstances in women’s lives. To do this, a larger number of pregnant participants is needed for reliable outcomes. Continuing validation and exploration of its predictive value may show if the PFC-PBI can be a screening tool or a tool to assess treatment-related improvement with regard to the psychological burden of women with PFC.

## Conclusion

The new Pelvic Floor Complaint-related Psychological Burden Inventory was found to be reliable and homogeneous with sufficient convergent and divergent validity. Therefore, it can be concluded that this new instrument is a valid and reliable instrument for use among women with PFC. Its predictive validity and its feasibility and diagnostic value for use in clinical practice need to be further examined in future research.

## Data Availability

OSF: The Pelvic Floor Complaint-related Psychological Burden Inventory. https://osf.io/dr7a6 ^28^ This project contains the following underlying data: 03-22-2023 CFA.omv: Split half data set for the confirmatory factor analysis 03-22-2023 PCA.omv: Split half data set for the principal component analysis 03-22-2023 PFC-PBI.omv: Complete data set for validation analysis This project contains the following extended data: 04-15-2023 Novel Questionnaire: 33 Dutch statements from the interviews Novel Questionnaire-English translation.docx Data are available under the terms of the Creative Commons Attribution 4.0 International license (CC-BY 4.0). The authors would like to thank all participants for their contribution to this study. Part of the participant recruitment was accomplished through a service paid by the first author to Hersenonderzoek.nl, a Dutch online registry that facilitates participant recruitment for neuroscience studies ( www.hersenonderzoek.nl). Hersenonderzoek.nl is funded by ZonMw-Memorabel (project no 73305095003), a project in the context of the Dutch Deltaplan Dementie, Gieskes-Stijbis Foundation, the Alzheimer’s Society in the Netherlands and Brain Foundation Netherlands.
