# Supplementary material for: Measuring the psychological burden of women with pelvic floor complaints: The psychometric characteristics of a new instrument
Source: Open Res Eur. 2024 Jun 3;3:83. Originally published 2023 May 17. [Version 2] doi: 10.12688/openreseurope.15833.2 (PMC11170070; doi:10.12688/openreseurope.15833.2)
Supplement: Peer review history

# Reviewer report: Reviewer response for version 2

**DOI:** 10.21956/openreseurope.19182.r41187

**Published:** 2024-06-12

**Authors:** Sule Gokyildiz Surucu

**Affiliations:** 1 Cukurova University, Adana, Turkey

In my opinion, the manuscript is appropriate to be accepted and indexed.

If applicable, is the statistical analysis and its interpretation appropriate?

Yes

Is the study design appropriate and is the work technically sound?

Yes

Is the work clearly and accurately presented and does it engage with the current literature?

Yes

Are the conclusions drawn adequately supported by the results?

Yes

Are sufficient details of methods and analysis provided to allow replication by others?

Yes

Are all the source data and materials underlying the results available?

Yes

Reviewer Expertise:

Pelvic floor health, sexual health

I confirm that I have read this submission and believe that I have an appropriate level of expertise to confirm that it is of an acceptable scientific standard.

# Reviewer report: Reviewer response for version 1

**DOI:** 10.21956/openreseurope.17104.r31750

**Published:** 2024-05-02

**Authors:** Kirstin R. Mitchell

**Affiliations:** 1 MRC/CSO Social and Public Health Sciences Unit, University of Glasgow, Glasgow, Scotland, UK

Thank you for the opportunity to review this interesting paper. I believe it makes an important contribution to accurate assessment of the psychological burden of pelvic floor complaints.

**Abstract:**

- Please include the key test statistics for the validity and reliability tests within the abstract.
- Plain language summary: Could you make clear here that the current research is a continuation of previous research by the same research group?

 

**Introduction:**

- The first introductory paragraph is very brief. As currently framed, the relevance of help-seeking is not very clear. I think you could do more to set out what measure gaps exist (and how this new measure will add to the field) and establish the rationale for why it is important to accurately measure the psychological burden. 
- It could be made clearer that the present study builds on - and is part of  - the same overall study by the same authorship team (refs. 20, 21). I think it would be good to have more details about conceptual development of the measure - possibly in the methods section?
- Conceptual mapping presumably means that women's lived experiences were included in the development of measure? This is a potential strength but is currently glanced over.  
- Para 2 – ‘because’ appears twice in same sentence. 

**Methods:**

- Who derived the 33 items? Were they cognitively tested by patients? Please describe.
- The sentence about the name of the PFC-PBI seems out of place. Could this go at the end of the methods section?
- The sentence on ethical approval appears twice - under data collection procedures and ethical considerations.
- I’m not qualified to comment in detail on the statistical approach, but I did wonder why you didn't include other criteria for item performance (e.g. non-response, aggregate adjacent endorsement, floor ceiling effects, face validity for participants) and why you were guided only by psychometric criteria and not by any theoretical considerations or looking for balance across your original domains?
- Also why were all items dropped in the preliminary phase on the basis of their correlation to the long list of items, when possibly they would have higher correlations once other items were removed? If the different types of distress were identified as important in your qualitative work, would that not be justification to include them even if they perform less well? It might due to poor wording of the item? Or might they have had higher item-total correlations if other items had been removed at the first stage?
- The RMSEA indicated a less acceptable fit, so is it fair to conclude excellent properties?

 

**Discussion:**

- The discussion of items that did not make it into the final measure seems based on the assumption that psychometric considerations trump theoretical ones (i.e. if the item does not correlate then it must not be relevant). There are some post-hoc explanations which seem based on the idea that the psychometric analysis must be correct. Is it possible that a different approach - e.g. eliminating items during both an EFA and CFA stage might have led to a different final set of items?
- Also, looking at the items themselves, could it be that the wording didn't sufficiently capture the idea of disappointment?

If applicable, is the statistical analysis and its interpretation appropriate?

I cannot comment. A qualified statistician is required.

Is the study design appropriate and is the work technically sound?

Yes

Is the work clearly and accurately presented and does it engage with the current literature?

Yes

Are the conclusions drawn adequately supported by the results?

Partly

Are sufficient details of methods and analysis provided to allow replication by others?

Yes

Are all the source data and materials underlying the results available?

Yes

Reviewer Expertise:

NA

I confirm that I have read this submission and believe that I have an appropriate level of expertise to confirm that it is of an acceptable scientific standard.

# Response

**Published:** 2024-05-07

**Authors:** Alma Brand

**Affiliations:** Psychology, Open Universiteit, Heerlen, Limburg, The Netherlands

We would like to thank reviewer Kirstin R. Mitchell kindly for the constructive feedback and recommendations.   We have responded to the indicated points to strengthen or clarify the text in the article. **Abstract:**

- The key statistics for the validity and reliability have been added.
- The plain language summary has been clarified.

**Introduction:**

- We added a rationale for measuring psychological burden.
- We clarified the point regarding research by the same authors.
- Some details about the conceptual development of the measure have been added to the methods section.
- The point made about women’s lived experiences being glanced over has been added.

**Method:**

- The point about the 33 statements has been clarified in the last paragraph of the introduction.
- The sentence about the name of the new instrument has been moved to the end of the methods section.
- The sentence about Ethical approval under the data collection procedures heading has been removed.
- We tried to clarify the origin of the 33 statements in the introduction section. We did not exclude theoretical considerations but built a theory first by performing the mixed-method studies, including items from all the emergent original clusters of distress in the conceptual model before performing the psychometric analysis on the items that emerged as internally consistent.
- Items were dropped in the preliminary phase in an iterative process to derive the most important and representative items of distress for the new instrument. In this study, the 33 pre-selected items from all the emergent types of distress in the conceptual model were included in the psychometric analysis. We have tried to clarify this in the last paragraph of the introduction and the method section under the internal consistency heading.
- We have reworded the pertinent text in the abstract. Thank you for noticing. The excellent properties mentioned in the results section under the CFA, refer to the internal consistency analyses of the CFA data sample. The RMSEA indicated a less acceptable fit, as is noted in this results section.

**Discussion:**

- Correlations if an item was deleted were evaluated, as well as the content of the (remaining) items throughout the whole process of elimination. During the PCA and CFA, no items emerged that needed to be eliminated. If so, we would have included this in the results. I am not sure if another procedure would have yielded another item pool because we purposely chose this method based on the results of the two mixed-method studies.
- Thank you for providing a word for what we intended to point out. We included your suggestion and clarified this at the end of the disappointment paragraph.

# Reviewer report: Reviewer response for version 1

**DOI:** 10.21956/openreseurope.17104.r32930

**Published:** 2023-07-20

**Authors:** Sule Gokyildiz Surucu

**Affiliations:** 1 Cukurova University, Adana, Turkey

The authors developed a new tool to determine the psychological burden of women with pelvic floor complaints. This article is based on two previous research of the authors ^1^^,^^2^. In the previous studies, limitations and difficulties experienced by women regarding pelvic floor complaints were evaluated, and a conceptual model of the psychological burden associated with pelvic floor complaints was developed. The lack of a tool in the literature to determine the psychological burdens associated with pelvic floor complaints and the development of such an assessment tool are the strengths of the study. I think, this paper addresses an important topic for women health and is a valuable addition to the literature. The scale will meet an important deficiency in this area.

 However, there are a few points in the article that need to be strengthened or clarified.

 There is an inconsistency between the method and the results sections. While it was stated in the method section that data were collected from women with and without pelvic floor complaints, only women with pelvic floor complaints were included in the results section.

 If "N" represents the number of items in Table 1, it would be more appropriate to explain the item as "I", which is the abbreviation of the item.

 One of the steps in the scale development process is to present the opinion of an expert who is knowledgeable in the content area who will review the item pool. Expert feedback are critical in the item generation and dimension identification process ^3^^,^^4^.Giving information about expert opinion in the study is recommended.

1. Brand AM, Waterink W, Stoyanov S, et al.: Restrictions and distress in daily, social, and sexual functioning, and intimate relationships in women with pelvic floor complaints: a mixed-method study. Health Care Women Int. 2022; 1–14.
2. Brand AM, Rosas S, Waterink W, et al.: Conceptualization and Inventory of the Sexual and Psychological Burden of Women With Pelvic Floor Complaints; A Mixed-Method Study. Sex Med. 2022; 10(3): 100504.
3. DeVellis RF. Scale Development: Theory and Applications Applied Social Research Methods SAGE Publications, 5. Ed. 2021.
4. Carpenter, S (2018). Ten steps in scale development and reporting: A guide for researchers. Communication methods and measures, 12(1), 25-44.

If applicable, is the statistical analysis and its interpretation appropriate?

Yes

Is the study design appropriate and is the work technically sound?

Yes

Is the work clearly and accurately presented and does it engage with the current literature?

Yes

Are the conclusions drawn adequately supported by the results?

Yes

Are sufficient details of methods and analysis provided to allow replication by others?

Yes

Are all the source data and materials underlying the results available?

Yes

Reviewer Expertise:

Pelvic floor health, sexual health

I confirm that I have read this submission and believe that I have an appropriate level of expertise to confirm that it is of an acceptable scientific standard, however I have significant reservations, as outlined above.

## References

[1] : Restrictions and distress in daily, social, and sexual functioning, and intimate relationships in women with pelvic floor complaints: a mixed-method study. *Health Care Women Int* .2022; 10.1080/07399332.2022.2062758 1-14 10.1080/07399332.2022.2062758 35471120

[2] : Conceptualization and Inventory of the Sexual and Psychological Burden of Women With Pelvic Floor Complaints; A Mixed-Method Study. *Sex Med* .2022;10(3) : 10.1016/j.esxm.2022.100504 100504 10.1016/j.esxm.2022.100504 35339057 PMC9177886

[3] : Scale Development: Theory and Applications Applied Social Research Methods. *SAGE Publications* .2021.;

[4] : Ten Steps in Scale Development and Reporting: A Guide for Researchers. *Communication Methods and Measures* .2018;12(1) : 10.1080/19312458.2017.1396583 25-44 10.1080/19312458.2017.1396583

# Response

**Published:** 2024-05-07

**Authors:** Alma Brand

**Affiliations:** Psychology, Open Universiteit, Heerlen, Limburg, The Netherlands

We would like to thank reviewer Sule Gokyildiz Surucu kindly for the constructive feedback and recommendations.   We have responded to the indicated points to strengthen or clarify the text in the article. **The inconsistency between method and results:** The first sentence of the results section has been replaced by:   Data from 552 Dutch women with PFC were included in the analyses because the instrument will be specifically used for assessment in this group of women. **The “N” in table 1:** Thank you for pointing this out. We have replaced “N” with “Nr. of item”. **Concerning the comment on expert opinions:** Thank you for this comment. We have included expert opinions as one of the steps in our scale development. We have done this a priori, within the Group Concept Mapping study that we refer to under reference nr. 21. This text has been added to the introduction section at the end of paragraph 2 for clarification: “In the conceptual study, expert opinions were included in the item selection process before the psychometric analysis was performed [21]”.
